# 

**Published:** 1873-10

**Authors:** 


					﻿The Soothern Medical Becord.
EDITED BY
T. S. POWELL, M. ID.,
AND
W. T. GOLDSMITH, M. JD.
ASSOCIATE] EDITORS.
GEORGIA.
Robert Battev, M. D..
R.	C. Word, AT. D.,
W. L. Kirkpatrick, AT. D.,
James A. Long. AL D..
W. L. AT. Harris. AT. D„
IT. L. Bartie. AL D._
W. C. Musgrove, M. D„
L. B. Bonchelle. At. D..
John C. Drake, AT. D.,
E. F. Knott. AT. D..
Thomas Bnrdell, AT. D..
E. II. AV. Hunter, AL D„
V.	S. Hitt. AT. D.,
J. E. Pope. AI. D..
J. A. Hunnicutt. AT. D..
A. H. Brantlev. Al D..
J. J Knott. AI. D..
J. 0. Wisdom AL D„
W.	W. Leake, AI. D.
NORTH CAROLINA.
J. R. Hughes. AL D._
S.	S. Satcliwell. AT. D.,
A. B. Pierce, AI. D.,
IT. Kelly. AL D..
fieo. A. Foote. AT. D.,
E. A. Anderson, AT. D.,
IT. T. Bahnson. AT. D.,
Willis Alston. AL D..
Thos. F. Wood. AT. D..
N. J. Pittman, AL D.
MISSISSIPPI.
C. B. Oallawav, AL D„
Edward Loa. M. I).,
S.	V. D. Hill. AI. D„
W. B. Harvev. AL D.,
J. W. AT. Shattuck. AL D.,
E.	T. Henry. AI. D .
T.	P Lockwood. Al. D..
Robert Kells, Al. D..
VIRGINIA.
Charles S. Lewis, M. D..
John II Claiborne. AL D.,
F.	Horner. Jr., AI. D.,
R.	J. Preston. Al D.,
A. S.J’avne. AL D.,
J. E. Lockridge. M. D.,
J. S. Wharton, AT. D.,
Wm. O. Owen. AL D..
Satn'l P. Ripley. AT. D..
Benj. Blackford. AT. D.,
E. A. Drewry. AL D.,
Rob B. Tunstall. AL D..
W. F. Barr. AT. D..
L. B. Anderson, AT. D..
S.	L. Jepson. Al. D., W. Va.,
J. AL Lazzell. AL I).
SOUTH CAROLINA.
E. T. McSwain. AI. D.,
G. Al. Rivers, Al. D,
TENNESSEE.
J. D. Tucker, AL D.,
Swan Al. Burnett, AI. D.
TEXAS.
S.	Al. Welch, M. D..
T.	J Heard. AL D.,
AV. A. Heard, M. D..
A. R. Kilpatiick, AI. D.
ARKANSAS.
A. D. nodge, AI D.,
J. K. Hodge. AI. D.
ALABAMA.
J. C. Knox. AI. I)..
Geo. F. Taylor. AL D.,
J. B. Barnett, AL D„
S. AL Hogan. AL D..
D. S. IIopping,.AL D..
.1 T. Searcv. AL I)..
S. F. Hill. AL D.
CORRESPONDING EDITORS :
Elv Van DeWarker. AL D.. New York.
John IL Weir. AT. D.. Illinois,
Tho«. J. Gallaher. AT. D.. Pennsylvania,
Robert Robson. AL D.. Indiana,
C. C. F. Gay. AL D.. New York.
Surgeon of Buffalo General Hospital.
W. T. Taylor. AL D.. Kentucky.
B. TV. Stone, AT. D.. Kentucky.
Assistant Phy-ician Western Lunatic Asylum.
James Tvson. AL I).. Philadelphia. Pa..
W. AV. L'een, AL I).. Philadelphia. Pa..
AI. IL Henrv. AT. D . New York.
Surgeon to N Y. Dispensary. Dep’t of
Venerial and Skin Diseases.
Wm. R. TVhitehead. AT. I).. Colorado Territory,
A. Patton, AT. D.. Indiana.
Benjamin Howard. Al. D.. New York.
Charles Kearns. AT. D.. Kentucky,
S.	C. Bussev. AL D., Washington, D. C.,
A. W. Ilobson. AL D„ Arkansas.
A. W. Calhoun. AL I). now Berlin. Prussia.
T.	Curtis Smith. AT. D , Ohio.
Geo. Strawbridge. AL D.. Philadelphia, Pa.
All business Letters should be addressed to POWELL & GOLDSMITH,
Post Office Box 527, Atlanta, Ga.
				

